# Calibration with or without phantom for fracture risk prediction in cancer patients with femoral bone metastases using CT-based finite element models

**DOI:** 10.1371/journal.pone.0220564

**Published:** 2019-07-30

**Authors:** Florieke Eggermont, Nico Verdonschot, Yvette van der Linden, Esther Tanck

**Affiliations:** 1 Orthopaedic Research Laboratory, Radboud Institute for Health Sciences, Radboud University Medical Center, Nijmegen, The Netherlands; 2 Laboratory of Biomechanical Engineering, University of Twente, Enschede, The Netherlands; 3 Department of Radiotherapy, Leiden University Medical Center, Leiden, The Netherlands; Universidad de Zaragoza, SPAIN

## Abstract

The objective of this study was to develop a new calibration method that enables calibration of Hounsfield units (HU) to bone mineral densities (BMD) without the use of a calibration phantom for fracture risk prediction of femurs with metastases using CT-based finite element (FE) models. Fifty-seven advanced cancer patients (67 femurs with bone metastases) were CT scanned atop a separate calibration phantom using a standardized protocol. Non-linear isotropic FE models were constructed based on the phantom calibration and on two phantomless calibration methods: the “air-fat-muscle” and “non-patient-specific” calibration. For air-fat-muscle calibration, peaks for air, fat and muscle tissue were extracted from a histogram of the HU in a standardized region of interest including the patient’s right leg and surrounding air. These CT peaks were linearly fitted to reference “BMD” values of the corresponding tissues to obtain a calibration function. For non-patient-specific calibration, an average phantom calibration function was used for all patients. FE failure loads were compared between phantom and phantomless calibrations. There were no differences in failure loads between phantom and air-fat-muscle calibration (p = 0.8), whereas there was a significant difference between phantom and non-patient-specific calibration (p<0.001). Although this study was not designed to investigate this, in four patients who were scanned using an aberrant reconstruction kernel, the effect of the different kernel seemed to be smaller for the air-fat-muscle calibration compared to the non-patient-specific calibration. With the air-fat-muscle calibration, clinical implementation of the FE model as tool for fracture risk assessment will be easier from a practical and financial viewpoint, since FE models can be made using everyday clinical CT scans without the need of concurrent scanning of calibration phantoms.

## Introduction

Patients with advanced cancer and bone metastases have an increased risk of a pathological fracture. Occurrence of these fractures in the femur of the patient leads to immediate reduced mobility, pain and distress, and causes reduced quality of life. When patients present with a painful femoral metastasis, treatment plans are based on the fracture risk estimated by the clinical team: patients with a low fracture risk undergo conservative treatment such as radiotherapy, while patients with a high fracture risk are considered for prophylactic stabilization surgery to reduce the chance of fracturing [1, 2]. In current clinical practice, fracture risk is estimated using CT scans and x-rays, but this appears to be difficult, leading to over and under treated patients [1].

Finite element (FE) models have shown to be promising as a tool for fracture risk prediction [3–7]. Quantitative Computed Tomography (QCT) scans can be used to segment patient-specific bone geometries that function as input for the FE models. Additionally, Hounsfield units (HU) in the QCT scan can be converted to bone mineral densities (BMD) that are used to model element-specific bone material properties [3–7]. Currently, these conversions to BMD are usually done with either solid or liquid calibration phantoms that contain certain known concentrations of for example calcium hydroxyapatite (CaCO_3_ or CaHA) or hydrogen dipotassium phosphate (K_2_HPO_4_ or KHP). These calibration phantoms are of reasonable size and need to be scanned along with the patient.

However, such separate calibration phantoms are not routinely available, quite expensive, and result in inability to use everyday clinical CT scans without a phantom for FE modeling. Since the FE patient databases are now dependent on CT scans including a calibration phantom made for scientific prospective studies, the databases are currently limited in size and clinical validation of these FE models moves slowly. If a phantomless calibration method is available to obtain BMD from HU, FE models can be generated retrospectively from clinical CT databases that lack calibration phantoms. In this manner, a large database could be built more easily for further validation of FE models for clinical fracture risk assessments in patients with femoral bone metastases. Additionally, a phantomless calibration method to use prospectively for each patient presenting with femoral bone metastases would be very helpful for clinical implementation of FE modeling as a fracture risk prediction tool.

Several methods have been developed for calibrating CT scans without a calibration phantom [8–15]. Some studies calibrate with the use of a calibration function obtained from a separate scan containing a calibration phantom [8], determine calibration factors based on CT scans that contain a calibration phantom and apply this calibration factor to CT scans without calibration phantoms [9], or calculate BMD using a regression model based on previous phantom calibration [15]. Another phantomless option is to use patient-specific internal calibration methods, which are based on HU of specific tissues, such as fat and muscle tissue [10–13] or external air and either aortic blood or visceral fat [14]. Studies comparing phantom calibration with phantomless calibrations showed that they yielded comparable results [8–15]. These studies used a single CT scanner or multiple CT scanners with a standardized protocol. Since it is known that changes in CT protocol can yield differences in HU [16–18], one could expect an effect of CT scanner or protocol for certain phantomless calibration methods on BMD or FE outcomes. Additionally, most studies only determined the effect of different calibration methods on vertebral trabecular BMD [8–12], but not on FE outcomes. The before mentioned calibration methods function well for trabecular BMD determination, probably because they cover HU in the same range of trabecular bone [10–14]. However, when applying these calibration methods to cortical bone far outside the range of calibration values, this could lead to extrapolation errors. Since FE models of femurs contain both trabecular and cortical bone material properties, it should be determined whether comparable results are obtained when using phantomless calibrations. Although both calibration with and without a phantom are based on linear relationships between HU and BMD, the non-linear relationship between BMD and bone material properties [19] probably will affect the fracture risk as calculated by the FE models in a non-linear manner. A study by Lee *et al*. tested the effect of a phantomless calibration based on external air and visceral fat on femoral FE strength using research-quality clinical-resolution CT scans that had been analyzed in prior clinical drug trials. They found good correspondence between phantom and phantomless calibrations, with a mean difference between the calibrations of 30 N (0.8%) [14]. Another study used a general calibration function to calibrate CT scans for femoral FE models and found errors of total strain energy of 0.91% in comparison with a phantom calibration [15].

Nevertheless, for FE modeling purposes, phantomless calibration methods have been studied limitedly. In addition, comparisons between several phantomless calibration methods for FE modeling have never been made.

The aim of this study was to develop a new calibration method that enables calibration of HU to BMD for finite element modeling of femurs with bone metastases without the use of a calibration phantom. The new phantomless calibration method should yield results similar to the phantom calibration. We evaluated two phantomless calibration methods: one based on HU of certain tissues within the CT scan and one non-patient-specific calibration function.

## Methods

### CT scans

Between August 2006 and September 2009 [7, 20], and January 2015 and April 2017, patients with cancer and bone metastases in the femur that were treated with radiotherapy in one of four radiotherapy institutes in the Netherlands (Radboud university medical center, Nijmegen; Leiden University Medical Center, Leiden; Radiotherapeutic Insitute Friesland, Leeuwarden; Bernard Verbeeten Institute, Tilburg) were asked to participate in a prospective cohort study that investigated if FE models were able to predict whether patients would or would not fracture their femur within six months. Hence, two cohorts were included both generated with an institutional review board approved research protocol (Regionale Toetsingscommissie Patiëntgebonden Onderzoek, Leeuwarden (NL12568.099.06) and Commissie Mensgebonden Onderzoek regio Arnhem–Nijmegen (2013/305)). All patients provided written informed consent. Institutes were instructed to generate QCT images of the patients using a standardized protocol, with the following settings: 120 kVp, 220 or variable mA, slice thickness 3 mm, pitch 1.5, spiral and standard reconstruction, field of view (FOV) 480 mm, in-plane resolution 0.9375 mm. In a few cases, the standardized protocol was accidentally violated, resulting in CT scans with a different FOV or reconstruction kernel. Since patients were included from four institutes, evidently four different CT scanners were used. These CT scanners comprised two Philips Brilliance Big Bore (Philips-1 and Philips-2, Philips Medical Systems, Eindhoven, The Netherlands) scanners, one GE Optima CT580 (GE Healthcare, Milwaukee, WI) and one Toshiba Aquilion/LB (Toshiba Medical Systems, Tokyo, Japan).

Patients with predominantly blastic femoral metastasis were excluded from the current study, as in a previous study [7], we found that the bone strength of such femurs was overestimated, probably due to unrealistically strong material properties in the FE model because of the high degree of mineralization in blastic lesions. Also, patients were excluded if they had a hip or knee prosthesis, the femur was incompletely scanned or the calibration phantom was not correctly placed, or body weight was absent from the clinical research files. This resulted in inclusion of 57 patients, with 67 femurs that were affected with bone metastases (Philips-1: 20 patients, 27 femurs; Philips-2: 16 patients, 18 femurs; GE: 8 patients, 8 femurs; Toshiba: 13 patients, 14 femurs).

### Phantom calibration

The patients were scanned on top of a solid calibration phantom (Image Analysis, Columbia, KY), that contained four known CaHA concentrations (0, 50, 100 and 200 mg/cm^3^). The known densities in this phantom were used to calibrate HU to CaHA density, which is a measure of BMD. A mean diaphyseal calibration was applied by determining the HU in the four rods over nine diaphyseal slices and correlate them with the known CaHA concentrations of the calibration phantom [7]. The selection of the diaphyseal slices was protocolized by starting with the slice containing no buttox or genitals and selecting the 8 consecutive slices. To correct for inter-scanner differences [16, 17, 21], the calibration curves were corrected toward the Philips-1 scanner, on which the FE model was validated [3, 6, 22], by cross-calibration. For this, we used phantom scans (Gammex 467 phantom, RMI Gammex, Middleton, WI, USA) [16] to determine the linear correlation function between aberrant CT scanner and the Philips-1 scanner (*HU_corrected_* = 0.998*HU+5.48 and *HU_corrected_* = 0.98*HU−3.32, for Philips-2 and Toshiba, respectively. GE was not corrected). Next, we applied this function to the HU within the calibration phantom and subsequently determined the corrected calibration function. In a similar manner, the CT scans acquired with a different reconstruction kernel were corrected (*HU_corrected_* = 0.97*HU+27.54 for Toshiba). CT scans with a different FOV were not corrected, as we have shown before that the effect on HU was negligible [17].

### Phantomless calibration

#### Air-fat-muscle calibration

We developed a phantomless calibration based on HU of certain tissues (air, fat, muscle and cortical tissue) within the CT scan. We first determined the accuracy of several calibrations with different combinations of air, fat, muscle and cortical tissue in a pilot study (see S1 File). The combination of air, fat and muscle yielded the highest correlation with respect to the phantom calibration, and was therefore selected as the first phantomless calibration method.

**Fig 1:**
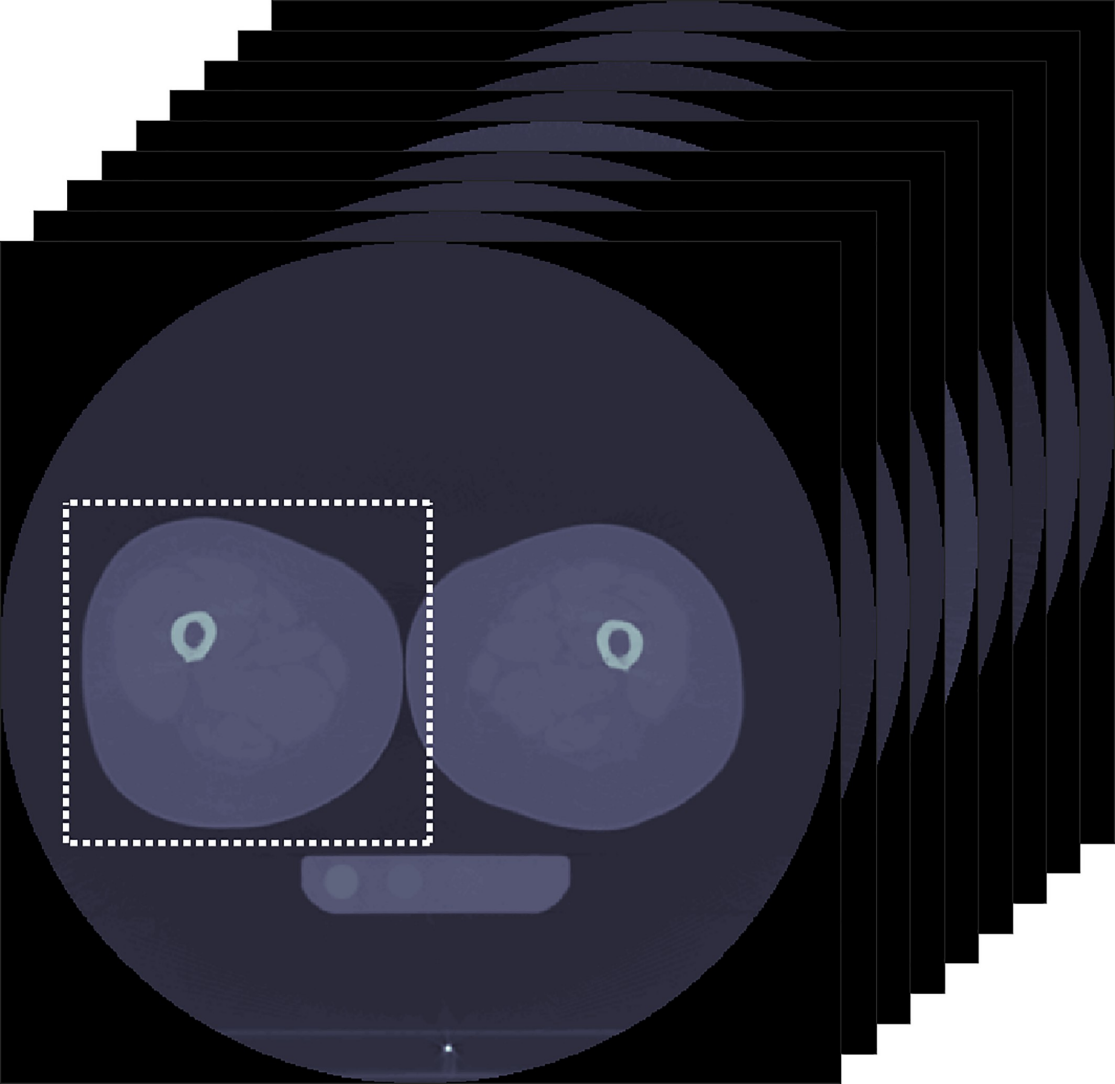
An example of the region of interest (white dashed box) over nine diaphyseal slices that was used for the phantomless air-fat-muscle calibration.

**Fig 2:**
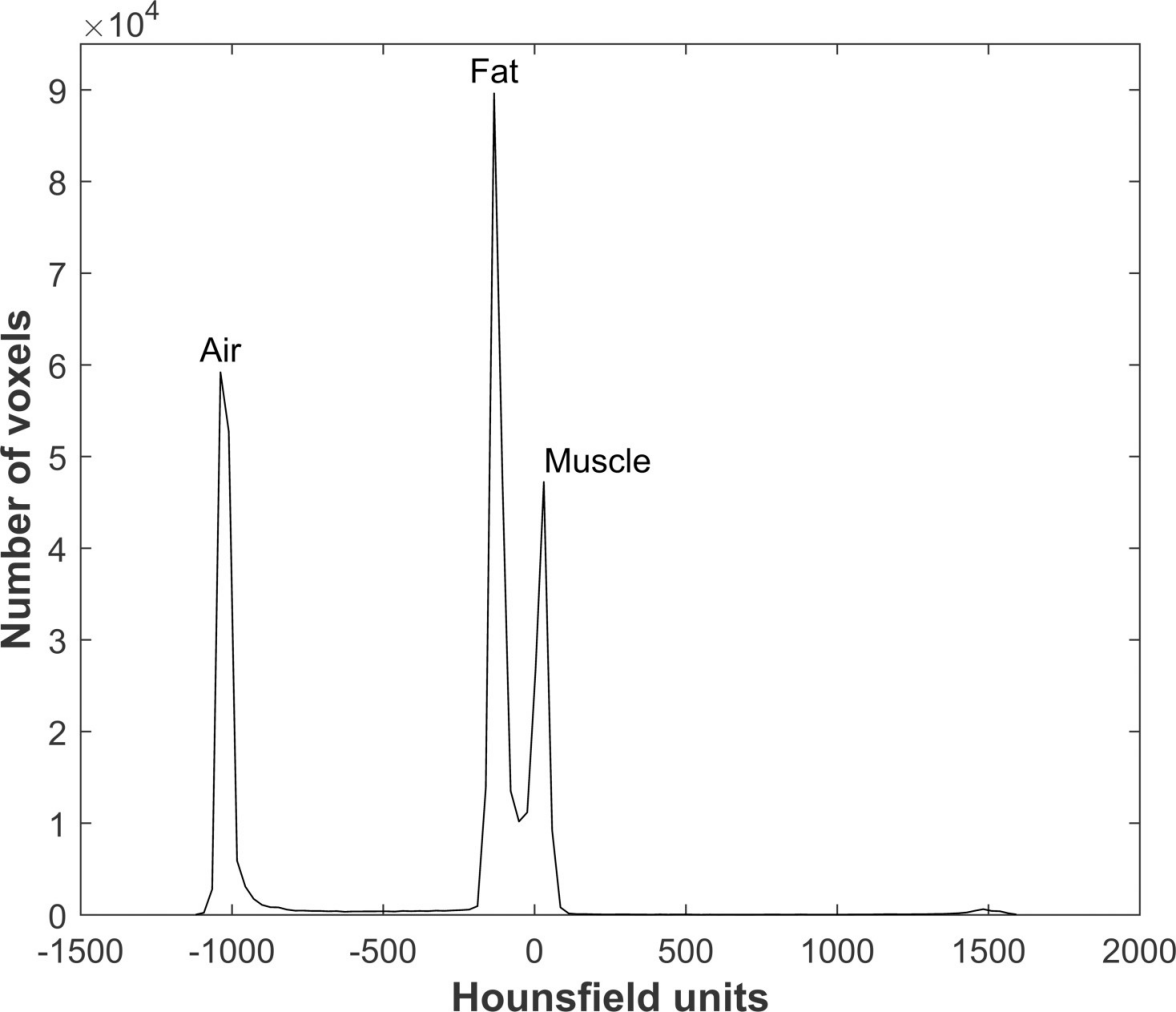
An example of a histogram of the Hounsfield units within the region of interest, used to extract the peaks for air, fat and muscle. An additional relatively small peak is visible around 1500 HU, indicating the cortical bone of the femur.

For the air-fat-muscle calibration, the same nine diaphyseal slices as used for the phantom calibration were selected, using the same protocol for slice selection. The succeeding steps of the air-fat-muscle calibration were completely automated. On the nine selected slices, a square region of interest was defined including the tissue of the right leg and some surrounding air (± 1 cm on each side of the leg; Fig 1). Next, a combined histogram of all HU in the volume of interest (i.e. nine slices together) was created to extract the peaks for air, fat and muscle tissue (Fig 2). The mode of the HU around the histogram peak (±50 HU) was calculated, to determine the exact peak in HU for each of the tissues. By using the mode, the method was least susceptible to outliers. Subsequently, the determined HU peaks were linearly fitted to the reference “BMD” values for each patient to obtain the air-fat-muscle calibration function. These values were obtained by phantom calibrating the HU peaks of air, fat and muscle of a randomized subgroup comprising 10 patients scanned on the Philips-1 scanner. Subsequently, we averaged and rounded them, resulting in reference “BMD” values of -840, -80 and 30 for air, fat and muscle, respectively. The linear fits between HU and “BMD” were very good with an average R^2^ of 1.000±0.000 (slope = 1.194±0.016, intercept = 2.232±12.042). No scanner- or kernel-specific correction was applied for the air-fat-muscle calibration method.

#### Non-patient-specific calibration

Additionally, we determined a non-patient-specific calibration function to convert HU to BMD by averaging all calibration functions of all 26 patients scanned on the Philips-1 scanner (6 patients were later excluded due to abovementioned reasons). We only used the 26 patients scanned on this particular CT scanner because of inter-scanner differences we found in previous studies [7, 16, 17]. The used patients were scanned on the CT scanner on which the FE model was validated [3, 6, 22]. This calibration function (*BMD* = 0.82*HU−4.2) was then applied to each of the 57 included patients. No scanner- or kernel-specific correction was applied for the non-patient-specific calibration method.

### FE models

FE models were created for each included femur according to a previously described protocol [3, 7]. These FE models have previously been validated in an experimental setting, by comparing experimental bone strength of cadaveric femurs with simulated lytic lesion with predicted bone strength by FE models [3, 6, 22]. These FE models have also shown to be able to predict fracture risk in a clinical setting [7]. In summary, the femoral geometry was obtained from the CT scans (Mimics 14.0, Materialise, Leuven, Belgium) and was converted to a solid mesh (Patran 2011, MSC Software Corporation, Santa Ana, CA, USA). Non-linear isotropic bone material properties [19] were calculated sequentially in three ways from the BMD that were obtained with 1) use of the calibration phantom, 2) the phantomless air-fat-muscle calibration method and 3) the non-patient-specific calibration method. For accurate comparison, only the material properties were varied, whereas the other aspects of the FE model remained unchanged. The FE model was positioned in a stance configuration by aligning the femoral head center with the knee joint center. The model was distally fixated by two bundles of high-stiffness springs and via a cup on the femoral head a displacement-driven load was applied. MSC.MARC (v2013.1, MSC Software Corporation, Santa Ana, CA, USA) was used for the FE simulations. We used Keyak’s material model to describe the post-failure behaviour, starting with an initial perfectly plastic phase, followed by a strain softening phase and an indefinite perfectly plastic phase [19]. Incremental displacement and contact normal forces were registered and it was assumed that fracture occurred when maximum total reaction force was reached, which was defined as the failure load. In total, 201 FE models were made (67 femurs × 3 calibration methods).

### Statistical analysis

The phantom calibration method was used as gold standard, as the FE model was validated with the use of this calibration method [3, 6, 22]. The mean failure loads and standard deviations (SD) for each of the calibration methods were determined to enable interpretation of the results that follow from the statistical analysis. A linear mixed model was used to determine the differences in failure load between the different calibration methods. Femur was nested within CT scanner, because patients were only scanned on one CT scanner each. Femur, nested in CT scanner, was added as random intercept to disregard the variability between femurs and CT scanners [17]. In this way, the model analyzes the effects of calibration methods, instead of differences between femurs. Although we initially did not intend to investigate this, it was additionally tested whether adding protocol violations (other FOV or reconstruction kernel) as a fixed factor resulted in a better model fit based on likelihood-ratio tests. Adding FOV as a fixed factor did not significantly improve the likelihood-ratio and was therefore not added, whereas reconstruction kernel did and was added to the model as fixed factor. The interaction between calibration method and kernel was added to the model, since it was significant and increased the model’s fit based on a likelihood-ratio test. The level of significance was defined at *p*<0.05. Additionally, we made the comparisons between the different calibration methods visible by creating correlation plots and Bland-Altman plots.

## Results

### Patients and CT scans

Sixty-seven femurs in 57 patients affected with bone metastases were included. Despite the protocolization of CT scanning, five femurs of five different patients scanned on two different CT scanners were scanned with an aberrant FOV (between 509 mm and 652 mm, instead of 480 mm). We found in a previous study that changes in FOV had little effect on failure loads [16, 17]. Additionally, on one of the CT scanners, four femurs of four different patients were scanned with a different reconstruction kernel (detail kernel for scanning of the head, instead of the standard bone kernel). As mentioned before, CT scans acquired with a different kernel were corrected.

### Differences between calibrations

The mean failure loads were 6.17 kN (SD 2.11 kN), 6.16 kN (SD 1.99 kN) and 5.95 kN (SD 2.01 kN) for the phantom calibration, air-fat-muscle calibration and non-patient-specific calibration, respectively.

**Fig 3:**
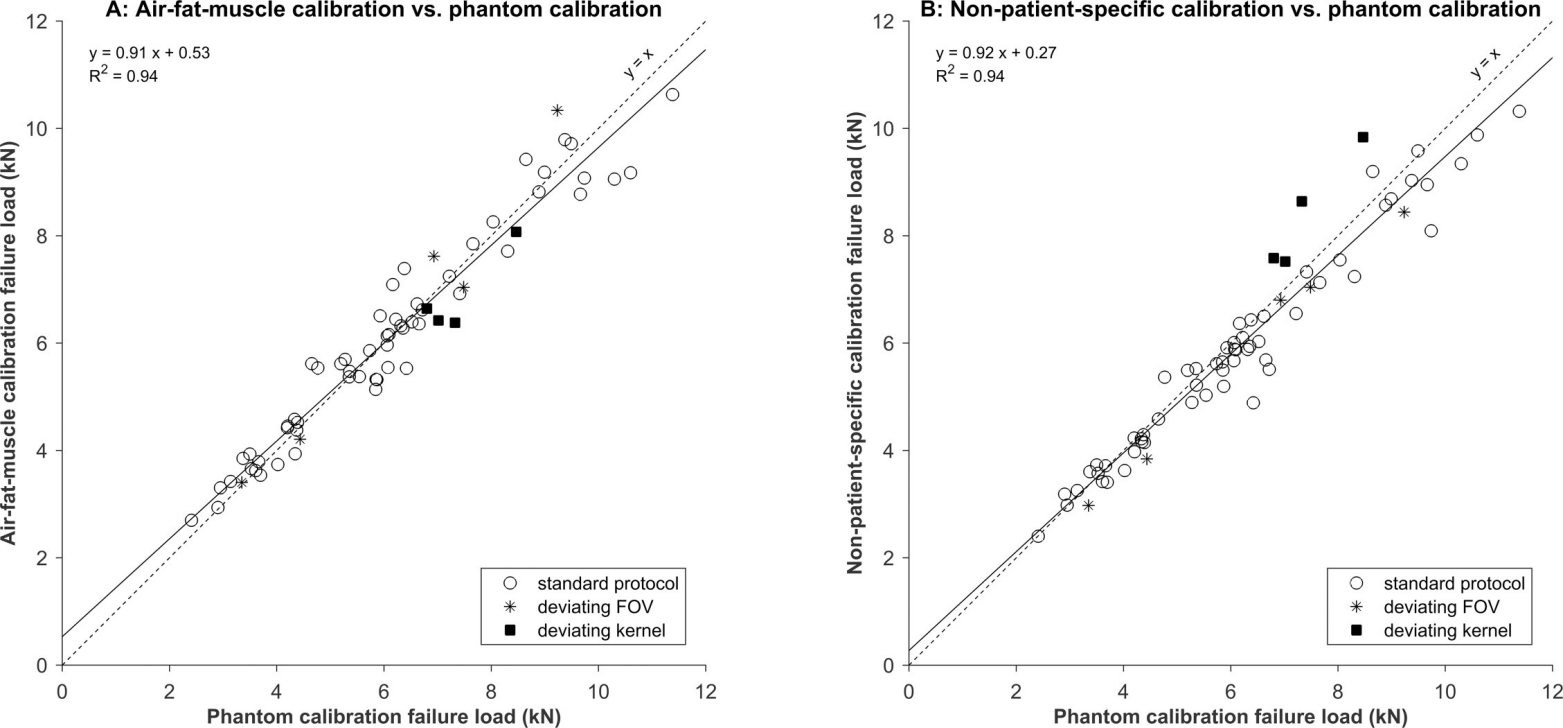
Correlations between phantom and air-fat-muscle calibration (A) and between phantom and non-patient-specific calibration (B).

**Fig 4:**
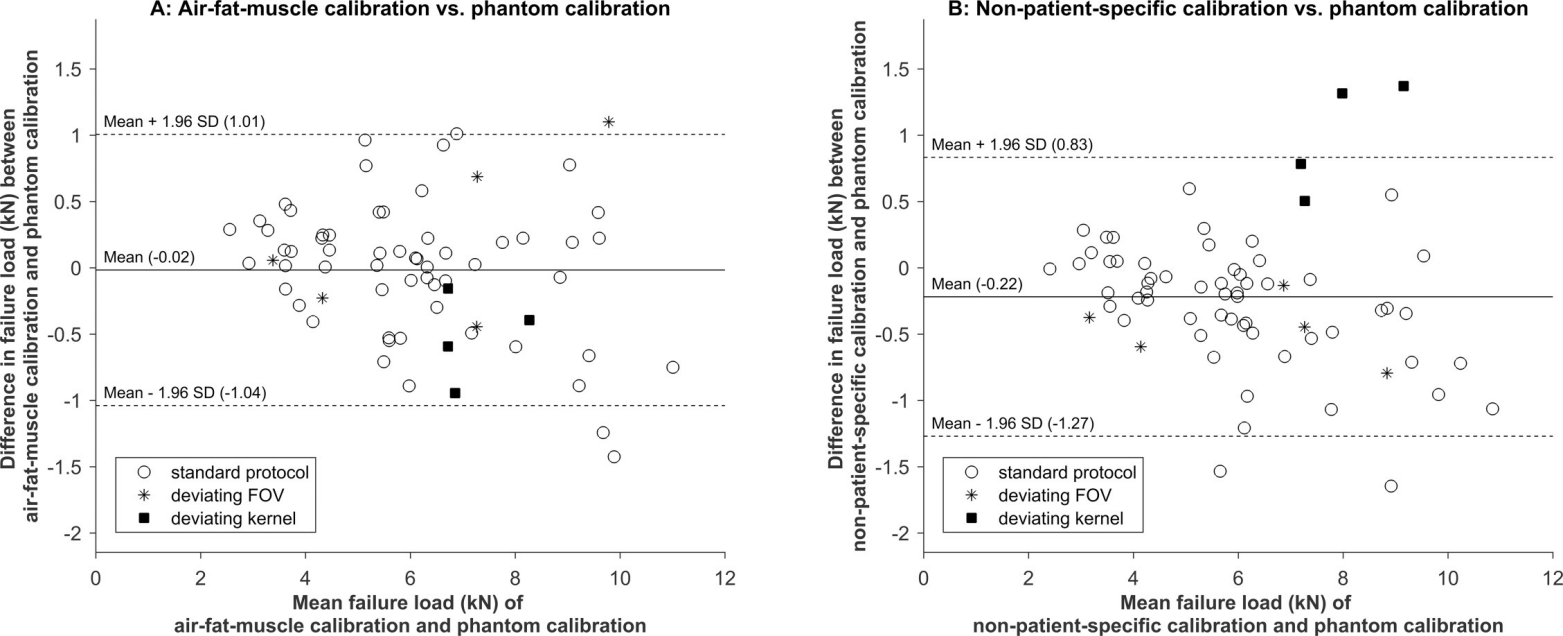
Bland-Altman plots for phantom versus air-fat-muscle calibration (A) and phantom versus non-patient-specific calibration (B).

The correlations between the phantom calibration and other calibrations were very high (R^2^ = 0.94 for air-fat-muscle calibration and R^2^ = 0.94 for non-patient-specific calibration, Fig 3). There were no significant differences in FE failure loads based on the phantom calibration and air-fat-muscle calibration (0.02 kN, 95% confidence interval (CI) -0.10–0.13 kN, p = 0.8), whereas the difference in failure loads between the phantom calibration and the non-patient-specific calibration was significant (-0.29 kN, 95% CI -0.41 –-0.18 kN, p < 0.001). Similarly, the Bland-Altman plots showed a slightly higher agreement between phantom and air-fat-muscle calibration (-0.02, 95% CI -1.04–1.01) compared to the agreement between phantom and non-patient-specific calibration (-0.22, 95% CI -1.27–0.83, Fig 4).

Changing reconstruction kernel had no significant effect on the phantom and air-fat-muscle calibration (1.31 kN, 95% CI -0.72–3.34 kN, p = 0.2 and 0.77 kN, 95% CI -1.26–2.8 kN, p = 0.5, respectively), whereas changing reconstruction kernel resulted in significantly higher failure loads when using the non-patient-specific calibration (2.60 kN, 95% CI 0.57–4.63 kN, p = 0.01).

Failure locations of the individual femurs were similar for the different calibration methods.

## Discussion

Previously, we developed a patient-specific FE model that can be used in clinical practice to differentiate between high or low fracture risk in advanced cancer patients with femoral bone metastases [7]. This FE model has been based on CT scans that are calibrated using a separate calibration phantom that is scanned together with the patient. The aim of this study was to develop and evaluate two phantomless calibration methods for FE modeling of femurs of patients with cancer and bone metastases.

We developed the air-fat-muscle calibration and the non-patient-specific calibration. We found strong correlations between the phantom calibration and both phantomless calibration methods. In addition, there was no significant difference in failure load between the phantom calibration and air-fat-muscle calibration, whereas the difference between phantom and non-patient-specific calibration was significant. Although the difference between the phantom and phantomless calibrations may seem small, it can be critical for patients that have failure loads around the threshold distinguishing patients with a low fracture risk from patients with a high fracture risk. Additionally, although this study was not designed to investigate this, it should be mentioned that the non-patient-specific calibration worked well for protocolized CT scans, but seemed to have trouble correcting for changes in reconstruction kernel. In those cases, the air-fat-muscle calibration was more accurate. Changes in FOV did not lead to differences in accuracy of the calibration methods, probably because changes in FOV have little effect on HU [16, 17]. When the same non-patient-specific calibration function is used for different CT protocols, this method lacks accuracy. This is inconvenient, since one would need to have a calibration function for each time a new CT scanner of protocol is being used. Therefore, a patient-specific calibration method, such as the air-fat-muscle calibration, would be more useful and robust.

Lee *et al*. tested a non-patient-specific phantomless calibration function on femoral FE models, and found it to be reliable as a replacement for phantom calibration [15]. However, they used one CT scanner and well protocolized CT scans, and therefore it has not yet been investigated whether non-patient-specific calibration methods are also useable for CT scans obtained on different CT scanners and with different settings. Additionally, another study investigated phantomless calibration for FE purposes and found comparable results between the phantom and phantomless calibrations [14]. They tested their phantomless air-fat method in 40 patients scanned on 24 different CT scanners, but only included scans that were made according to a standard scan protocol. Other studies used combinations of air and fat [14] or fat and muscle [10–13] for their phantomless calibrations, but none used the combination of air, fat and muscle, like we did. In a pilot study (S1 File), we first determined the accuracy of several calibrations with different combinations of air, fat, muscle and cortical tissue, but we found that the combination of air, fat and muscle yielded the highest correlation with respect to the phantom calibration. Addition of cortical tissue did not to improve the correlation, which can be explained by the large variation in cortical density between patients.

In a number of other studies small ROIs were placed within certain tissues by hand to obtain the reference HU for the calibration [11–13], which can be susceptible to inter-observer errors. We limited the variation between possible observers by automating all calibration methods. The only step that required manual input was the selection of the CT slices used for the calibrations. However, slice selection was strictly protocolized as well.

Each calibration method has its advantages and disadvantages. Calibration based on a calibration phantom that is scanned along with the patient has the benefit that the phantom is similarly affected as the FE modeled bone itself by patient- and scan-specific characteristics or artifacts, such as beam hardening, and might therefore be able to correct for such scan-specific characteristics or artifacts. The air-fat-muscle calibration method may be even better in capturing such potential artifacts, since the tissues on which the calibration is based are closer to the FE modeled bone compared to a calibration phantom. The non-patient-specific calibration cannot correct for any patient-specific CT artifacts.

Another important advantage of a calibration phantom is that the calcium densities in this phantom are precisely known. However, these usually only comprise low calcium densities, since high densities in the calibration phantom can lead to unnecessary artifacts in patient CT scans. As a result, the low calcium densities in the calibration phantom have to be extrapolated to higher (cortical) bone-equivalent densities, which is susceptible to errors. That also applies to the air-fat-muscle calibration.

Furthermore, we have seen that calibration phantoms can be affected by shadow artifacts caused by air gaps between patient and calibration phantom, leading to errors in the calibration [7, 16, 17]. Such shadow artifacts are not relevant for the air-fat-muscle calibration. However, possible patient-specific variations in fat or muscle composition can affect the air-fat-muscle calibration, mainly when a patient is suffering from pathologies that are known to affect the attenuation values on CT [23]. We assume that variations will usually be small, since we used the mode instead of mean or median of the HU peaks. Taking air as reference should be without problems, as the radiodensity of air is also used for calibration of attenuation coefficients to HU. The main disadvantage of the patient-specific calibration involves the fact that HU can vary between different CT scanners [16, 17, 24–26], and therefore it might be better to use scanner-specific calibration functions, as mentioned before. However, this requires generating new calibration functions each time a new scanner is used, which is quite labor-intensive.

Moreover, the major downsides of using separate calibration phantoms in clinical practice are the fact that patients have to lie on top of a separate mattress, and for the departments the expensive price and the logistical challenges it brings to scan each patient using the phantom. Additionally, when using the phantom calibration, there is a need for a scanner- and kernel-specific correction, for which extra CT scans of a tissue characterizing phantom have to be made [16, 17]. This requires CT scanning and analyzing of many extra CT scans, mainly when one would want corrections for all different reconstruction kernels, which is hardly workable. Both the air-fat-muscle as the non-patient-specific calibration do not require additional logistics or costs. Also, the air-fat-muscle calibration seems to be less affected by changes in CT scanner or protocols, possibly because it uses reference tissues that are closer to the FE modeled bone in the isocenter. It is known that CT scans are affected by scanner and settings in a non-uniform manner, with a different effect near the isocenter of the FOV in comparison to the edges of the FOV, where the calibration phantom is placed. On the contrary, using the same non-patient-specific calibration function on all CT scanners and for all CT protocols, it will not be able to supply any form of correction. As a result, air-fat-muscle calibration seemed to be preferable over non-patient-specific calibration, as the air-fat-muscle calibration seemed to be better in handling deviations to the scan protocol comparable to the phantom calibration.

It should be mentioned that there was no gold standard while evaluating the different calibration methods. As the FE model has been validated while making use of a phantom calibration [3], we chose this method to be the gold standard. This validation was done by creating FE models of cadaveric femurs, which were experimentally loaded until failure, and correlating the predicted failure load with the experimental failure load [3]. Although it is impossible to achieve this, it would be better to know the real failure loads of the patients’ femurs. Then it would be possible to investigate which of all calibration methods is the best in approaching the true failure loads.

In conclusion, phantomless calibration of CT scans using the air-fat-muscle calibration method is preferable over the non-patient-specific calibration method. The phantomless calibration method will stimulate the prospective use of the FE model as a fracture risk prediction tool for each patient that presents with femoral bone metastases with clinical implementation as ultimate goal. Additionally, with the use of the phantomless calibration method, FE models of retrospective CT scans without calibration phantoms can be generated and a large database can be built that can be used for the validation of FE models for application to fracture risk assessment in patients with femoral bone metastases.

## Supporting information

S1 Dataset All relevant data. (PDF)

S1 File Pilot study to determine the most accurate phantomless calibration method. (PDF)

## References

[1] Van der Linden YM, Dijkstra PD, Kroon HM, Lok JJ, Noordijk EM, Leer JW, et al Comparative analysis of risk factors for pathological fracture with femoral metastases. J Bone Joint Surg Br. 2004;86(4):566–73. . 15174555

[2] Van der Linden YM, Kroon HM, Dijkstra SP, Lok JJ, Noordijk EM, Leer JW, et al Simple radiographic parameter predicts fracturing in metastatic femoral bone lesions: results from a randomised trial. Radiother Oncol. 2003;69(1):21–31. . 14597353 10.1016/s0167-8140(03)00232-9

[3] Derikx LC, van Aken JB, Janssen D, Snyers A, van der Linden YM, Verdonschot N, et al The assessment of the risk of fracture in femora with metastatic lesions: comparing case-specific finite element analyses with predictions by clinical experts. J Bone Joint Surg Br. 2012;94(8):1135–42. Epub 2012/07/31. 10.1302/0301-620X.94B8.28449 . 22844058

[4] Goodheart JR, Cleary RJ, Damron TA, Mann KA. Simulating activities of daily living with finite element analysis improves fracture prediction for patients with metastatic femoral lesions. J Orthop Res. 2015;33(8):1226–34. 10.1002/jor.22887 . 25761000

[5] Keyak JH, Kaneko TS, Rossi SA, Pejcic MR, Tehranzadeh J, Skinner HB. Predicting the strength of femoral shafts with and without metastatic lesions. Clin Orthop. 2005;439:161–70. 10.1097/01.blo.0000174736.50964.3b . 16205155

[6] Tanck E, van Aken JB, van der Linden YM, Schreuder HW, Binkowski M, Huizenga H, et al Pathological fracture prediction in patients with metastatic lesions can be improved with quantitative computed tomography based computer models. Bone. 2009;45(4):777–83. Epub 2009/06/23. 10.1016/j.bone.2009.06.009 . 19539798

[7] Eggermont F, Derikx LC, Verdonschot N, Van der Geest ICM, De Jong MAA, Snyers A, et al Can patient-specific finite element models better predict fractures in metastatic bone disease than experienced clinicians? Towards introducing computational modelling into daily clinical practice. Bone Joint Res. 2018;7(6):430–9. 10.1302/2046-3758.76.BJR-2017-0325.R2 30034797 PMC6035356

[8] Habashy AH, Yan X, Brown JK, Xiong X, Kaste SC. Estimation of bone mineral density in children from diagnostic CT images: a comparison of methods with and without an internal calibration standard. Bone. 2011;48(5):1087–94. Epub 2010/12/28. 10.1016/j.bone.2010.12.012 21185418 PMC4780050

[9] Budoff MJ, Malpeso JM, Zeb I, Gao YL, Li D, Choi TY, et al Measurement of phantomless thoracic bone mineral density on coronary artery calcium CT scans acquired with various CT scanner models. Radiology. 2013;267(3):830–6. Epub 2013/02/27. 10.1148/radiol.13111987 . 23440323

[10] Boden SD, Goodenough DJ, Stockham CD, Jacobs E, Dina T, Allman RM. Precise measurement of vertebral bone density using computed tomography without the use of an external reference phantom. J Digit Imaging. 1989;2(1):31–8. 10.1007/bf03168013 . 2488150

[11] Mueller DK, Kutscherenko A, Bartel H, Vlassenbroek A, Ourednicek P, Erckenbrecht J. Phantom-less QCT BMD system as screening tool for osteoporosis without additional radiation. Eur J Radiol. 2011;79(3):375–81. Epub 2010/03/13. 10.1016/j.ejrad.2010.02.008 . 20223609

[12] Weaver AA, Beavers KM, Hightower RC, Lynch SK, Miller AN, Stitzel JD. Lumbar Bone Mineral Density Phantomless Computed Tomography Measurements and Correlation with Age and Fracture Incidence. Traffic injury prevention. 2015;16 Suppl 2:S153–60. Epub 2015/10/06. 10.1080/15389588.2015.1054029 26436225 PMC4602406

[13] Boomsma MF, Slouwerhof I, van Dalen JA, Edens MA, Mueller D, Milles J, et al Use of internal references for assessing CT density measurements of the pelvis as replacement for use of an external phantom. Skeletal Radiol. 2015;44(11):1597–602. Epub 2015/07/16. 10.1007/s00256-015-2206-5 . 26173417

[14] Lee DC, Hoffmann PF, Kopperdahl DL, Keaveny TM. Phantomless calibration of CT scans for measurement of BMD and bone strength-Inter-operator reanalysis precision. Bone. 2017;103:325–33. Epub 2017/08/06. 10.1016/j.bone.2017.07.029 28778598 PMC5636218

[15] Lee YH, Kim JJ, Jang IG. Patient-Specific Phantomless Estimation of Bone Mineral Density and Its Effects on Finite Element Analysis Results: A Feasibility Study. Computational and mathematical methods in medicine. 2019;2019:4102410 Epub 2019/02/06. 10.1155/2019/4102410 30719069 PMC6335860

[16] Free J, Eggermont F, Derikx L, Van Leeuwen R, Van der Linden Y, Jansen W, et al The effect of different CT scanners, scan parameters and scanning setup on Hounsfield units and calibrated bone density: a phantom study. Biomedical Physics & Engineering Express. 2018;4(055013).

[17] Eggermont F, Derikx LC, Free J, Van Leeuwen R, Van der Linden YM, Verdonschot N, et al Effect of Different CT Scanners and Settings on Femoral Failure Loads Calculated by Finite Element Models J Orthop Res. 2018 10.1002/jor.23890 29508905 PMC6120464

[18] Giambini H, Dragomir-Daescu D, Huddleston PM, Camp JJ, An KN, Nassr A. The Effect of Quantitative Computed Tomography Acquisition Protocols on Bone Mineral Density Estimation. J Biomech Eng. 2015;137(11). Artn 114502 10.1115/1.4031572 ISI:000362842900010. 26355694 PMC4844109

[19] Keyak JH, Kaneko TS, Tehranzadeh J, Skinner HB. Predicting proximal femoral strength using structural engineering models. Clin Orthop. 2005;(437):219–28. Epub 2005/08/02. 10.1097/01.blo.0000164400.37905.22 . 16056052

[20] Eggermont F, Derikx LC, Verdonschot N, Hannink G, Kaatee R, Tanck E, et al Limited short-term effect of palliative radiation therapy on quantitative computed tomography-derived bone mineral density in femora with metastases. Adv Radiat Oncol. 2017;2(1):53–61. Epub 2017/07/26. 10.1016/j.adro.2016.11.001 28740915 PMC5514233

[21] Carpenter RD, Saeed I, Bonaretti S, Schreck C, Keyak JH, Streeper T, et al Inter-scanner differences in in vivo QCT measurements of the density and strength of the proximal femur remain after correction with anthropomorphic standardization phantoms. Med Eng Phys. 2014;36(10):1225–32. Epub 2014/07/09. 10.1016/j.medengphy.2014.06.010 . 25001172 PMC4589175

[22] Derikx LC, Vis R, Meinders T, Verdonschot N, Tanck E. Implementation of asymmetric yielding in case-specific finite element models improves the prediction of femoral fractures. Comput Methods Biomech Biomed Engin. 2011;14(2):183–93. Epub 2011/02/22. 10.1080/10255842.2010.542463 . 21337224

[23] Aubrey J, Esfandiari N, Baracos VE, Buteau FA, Frenette J, Putman CT, et al Measurement of skeletal muscle radiation attenuation and basis of its biological variation. Acta physiologica. 2014;210(3):489–97. Epub 2014/01/08. 10.1111/apha.12224 24393306 PMC4309522

[24] Birnbaum BA, Hindman N, Lee J, Babb JS. Multi-detector row CT attenuation measurements: assessment of intra- and interscanner variability with an anthropomorphic body CT phantom. Radiology. 2007;242(1):109–19. Epub 2006/12/23. 10.1148/radiol.2421052066 . 17185663

[25] Levi C, Gray JE, McCullough EC, Hattery RR. The unreliability of CT numbers as absolute values. AJR Am J Roentgenol. 1982;139(3):443–7. 10.2214/ajr.139.3.443 . 6981306

[26] Sande EP, Martinsen AC, Hole EO, Olerud HM. Interphantom and interscanner variations for Hounsfield units—establishment of reference values for HU in a commercial QA phantom. Phys Med Biol. 2010;55(17):5123–35. 10.1088/0031-9155/55/17/015 . 20714048

