# Supplementary material for: Calibration with or without phantom for fracture risk prediction in cancer patients with femoral bone metastases using CT-based finite element models
Source: PLoS One. 2019 Jul 30;14(7):e0220564. doi: 10.1371/journal.pone.0220564 (PMC6667162; doi:10.1371/journal.pone.0220564)
Supplement: S1 Dataset — (PDF) [file pone.0220564.s001.pdf]

S1 Dataset. All relevant data

Patient	Side	BW	Phantom calibration	Air-fat-muscle calibration	Non-patient-specific calibration	Scanner	FOV	Kernel
01	L	56	5196	5617	5494	Philips-1	480	standard
02	L	73	3373	3854	3605	Philips-1	480	standard
02	R	73	3500	3934	3731	Philips-1	480	standard
03	L	55	6620	6731	6500	Philips-1	480	standard
03	R	55	3527	3662	3575	Philips-1	480	standard
04	L	73	4333	4580	4218	Philips-1	480	standard
04	R	73	2950	3305	2982	Philips-1	480	standard
05	R	48	4200	4424	4233	Philips-1	480	standard
06	L	85	9492	9715	9581	Philips-1	480	standard
07	L	90	6221	6445	6104	Philips-1	480	standard
08	L	57	6655	6357	5687	Philips-1	480	standard
08	R	57	7660	7852	7129	Philips-1	480	standard
09	R	84	6317	6323	5883	Philips-1	480	standard
10	L	57	6092	6161	5877	Philips-1	480	standard
10	R	57	5928	6510	5916	Philips-1	480	standard
11	R	108	5854	5327	5498	Philips-1	480	standard
12	L	90	4768	5539	5366	Philips-1	480	standard
13	R	80	10298	9056	9343	Philips-1	480	standard
14	R	89	8891	8820	8571	Philips-1	480	standard
15	R	50	2903	2938	3188	Philips-1	480	standard
16	L	65.5	5735	5860	5618	Philips-1	480	standard
16	R	65.5	3666	3790	3716	Philips-1	480	standard
17	R	62.7	6075	5545	5886	Philips-1	480	standard
18	L	76	7414	6923	7327	Philips-1	480	standard
18	R	76	8993	9185	8688	Philips-1	480	standard
19	L	63	4373	4381	4294	Philips-1	480	standard
20	R	71	5353	5372	5526	Philips-1	480	standard
21	L	84	9235	10336	8441	Philips-2	612	standard
22	R	92.5	4437	4210	3843	Philips-2	638	standard
23	R	87	3349	3407	2976	Philips-2	652	standard
24	R	95	8036	8261	7551	Philips-2	480	standard
25	R	65	9738	9076	8093	Philips-2	480	standard
26	R	94	9374	9791	9030	Philips-2	480	standard
27	R	65	5276	5698	4895	Philips-2	480	standard
28	R	65	11382	10632	10319	Philips-2	480	standard
29	R	71	6354	6280	5939	Philips-2	480	standard
30	L	79	6523	6396	6030	Philips-2	480	standard
31	R	84	5541	5376	5030	Philips-2	480	standard
32	L	75	6165	7092	6367	Philips-2	480	standard
32	R	75	6379	7391	6434	Philips-2	480	standard
33	L	63	4653	5618	4587	Philips-2	480	standard
34	L	55	2410	2700	2403	Philips-2	480	standard
35	L	82	7220	7245	6551	Philips-2	480	standard
36	L	95	6421	5532	4888	Philips-2	480	standard
36	R	95	6719	6621	5512	Philips-2	480	standard
37	L	79	8308	7714	7241	GE	480	standard
38	R	57	5362	5474	5218	GE	480	standard
39	R	75	4022	3739	3626	GE	480	standard
40	L	81	6059	5966	5673	GE	480	standard
41	L	65	3609	3625	3421	GE	480	standard
42	R	54	4390	4525	4148	GE	480	standard
43	L	76	5846	5139	5650	GE	480	standard
44	L	92	5869	5321	5196	GE	480	standard
45	R	75	6801	6645	7585	Toshiba	480	detail
46	L	85.5	8467	8073	9838	Toshiba	480	detail
47	L	90	6933	7621	6800	Toshiba	535	standard
48	L	73	6062	6137	6011	Toshiba	480	standard
49	L	75.5	7325	6381	8641	Toshiba	480	detail
50	R	88	7016	6424	7521	Toshiba	480	detail
51	L	69	4344	3939	4162	Toshiba	480	standard
52	L	90	3698	3538	3408	Toshiba	480	standard
53	L	56	3140	3423	3255	Toshiba	480	standard
54	L	75	4206	4454	3978	Toshiba	480	standard
55	L	56	10599	9175	9879	Toshiba	480	standard
55	R	56	9663	8775	8952	Toshiba	480	standard
56	L	67	8648	9425	9198	Toshiba	480	standard
57	R	96	7487	7043	7041	Toshiba	509	standard
