# Supplementary material for: Calibration with or without phantom for fracture risk prediction in cancer patients with femoral bone metastases using CT-based finite element models
Source: PLoS One. 2019 Jul 30;14(7):e0220564. doi: 10.1371/journal.pone.0220564 (PMC6667162; doi:10.1371/journal.pone.0220564)
Supplement: S1 File — (PDF) [file pone.0220564.s002.pdf]

S1 File. Pilot study to determine the most accurate phantomless calibration method

For the drafting of the phantomless calibration, we first performed an extensive literature search to determine what methods were already being used. We found that calibration based on combinations of fat and muscle tissue [1-4] or external air and either aortic blood or visceral fat [5] were used for vertebral trabecular BMD measurements.

Subsequently, peaks for air, fat, muscle and cortical tissue were subtracted from the patients' CT scans. Nine diaphyseal slices were selected, starting with the slice containing no buttox or genitals and selecting the 8 consecutive slices. On the nine selected slices, a square region of interest was defined including the tissue of the right leg and some surrounding air (± 1 cm on each side of the leg). Next, a histogram of the HU in this region was created to extract the peaks for air, fat and muscle tissue. The mode of the HU around the histogram peak (±50 HU) was calculated, to determine the exact peak in HU for each of the tissues. The determined HU peaks were linearly correlated to fixed "BMD" values of -840, -80, 30 and 1210 for air, fat, muscle and cortical tissue, respectively. The fixed "BMD" values were obtained by phantom calibrating the HU peaks of air, fat, muscle and cortical bone of a randomized subgroup comprising 10 patients scanned on the Philips-1 scanner, and subsequently averaging and rounding them.

For this pilot, we decided to continue with air-fat-muscle (AFM, because this method yielded the highest correlation with phantom calibration), fat-muscle (FM, because this method is used for BMD measurements in most studies) and air-fat-muscle-cortex (AFMC, because this method includes the largest range of HU and would avoid extrapolation of the calibration to the higher densities in cortical bone).

For a subselection of patients (n = 40), FE models were created using these phantomless calibration methods to determine the correlation with phantom calibration in terms of failure load (see Figure below). Since the AFM calibration resulted in the largest correlation (R^2 = 0.95, see Figure B), we chose to use this method as phantomless calibration.

Figure: Correlations between phantom and air-fat-muscle-cortex (AFMC, figure A), air-fat-muscle (AFM, figure B) and fat-muscle (FM, figure C) calibrations.

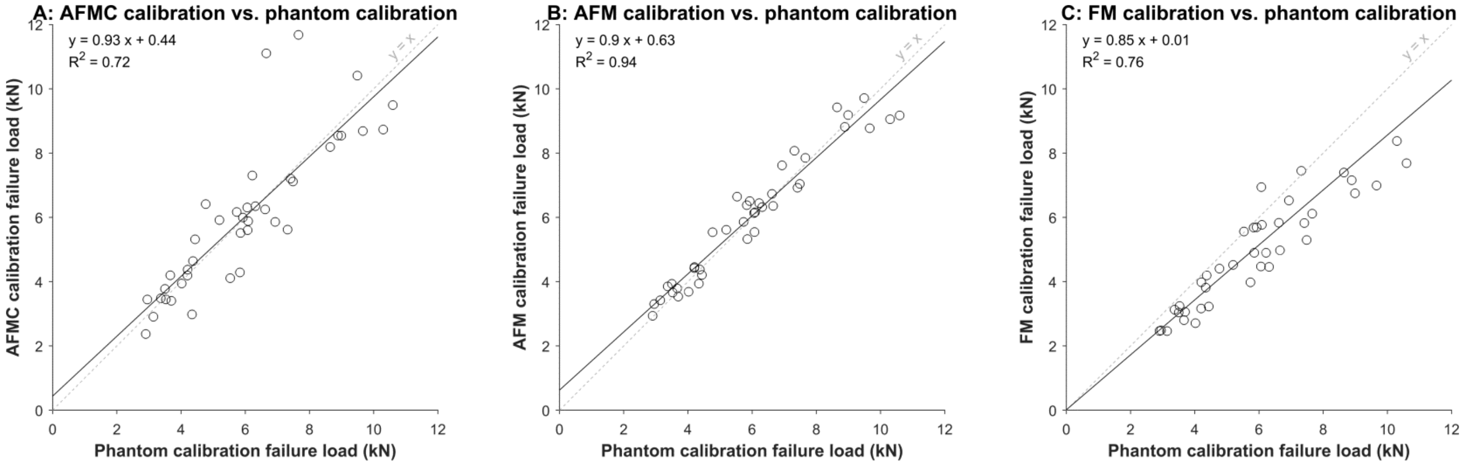

References

1. Boden SD, Goodenough DJ, Stockham CD, Jacobs E, Dina T, Allman RM. Precise measurement of vertebral bone density using computed tomography without the use of an external reference phantom. J Digit Imaging. 1989;2(1):31-8.
2. Mueller DK, Kutscherenko A, Bartel H, Vlassenbroek A, Ourednicek P, Erckenbrecht J. Phantom-less QCT BMD system as screening tool for osteoporosis without additional radiation. Eur J Radiol. 2011;79(3):375-81.
3. Weaver AA, Beavers KM, Hightower RC, Lynch SK, Miller AN, Stitzel JD. Lumbar Bone Mineral Density Phantomless Computed Tomography Measurements and Correlation with Age and Fracture Incidence. Traffic injury prevention. 2015;16 Suppl 2:S153-60.
4. Boomsma MF, Slouwerhof I, van Dalen JA, Edens MA, Mueller D, Milles J, et al. Use of internal references for assessing CT density measurements of the pelvis as replacement for use of an external phantom. Skeletal Radiol. 2015;44(11):1597-602.
5. Lee DC, Hoffmann PF, Kopperdahl DL, Keaveny TM. Phantomless calibration of CT scans for measurement of BMD and bone strength-Inter-operator reanalysis precision. Bone. 2017;103:325-33.
